# Serum neurofilament light chain and glial fibrillary acidic protein predicting multiple sclerosis after clinically isolated syndrome

**DOI:** 10.1007/s00415-026-13667-7

**Published:** 2026-02-09

**Authors:** Cato E. A. Corsten, Veerle S. A. Geraedts, Ana M. Marques, Marie-José Melief, Barry Koelewijn - van Vliet, Jeroen van Rooij, Marcello Ciaccio, Luisa Agnello, Jens Kuhle, Andrei N. Tintu, Beatrijs Wokke, Joost Smolders

**Affiliations:** 1 Department of Neurology, MS Center ErasMS, Erasmus MC University Medical Center, Rotterdam, The Netherlands; 2 Department of Immunology, MS Center ErasMS, Erasmus MC University Medical Center, Rotterdam, The Netherlands; 3 Department of Clinical Chemistry, Erasmus MC University Medical Center, Rotterdam, The Netherlands; 4 Department of Internal Medicine, Erasmus MC University Medical Center, Rotterdam, The Netherlands; 5 Department of Biomedicine, Neurosciences and Advanced Diagnostics, Institute of Clinical Biochemistry, Clinical Molecular Medicine and Clinical Laboratory Medicine, University of Palermo, Palermo, Italy; 6 Departments of Clinical Research and Biomedicine, Research Centre for Clinical Neuroimmunology and Neuroscience, Multiple Sclerosis Centre, Neurology, University of Basel and University Hospital Basel, Basel, Switzerland; 7 Neuroimmunology Research Group, Netherlands Institute for Neuroscience, Amsterdam, The Netherlands

**Keywords:** Biomarker, Multiple sclerosis, Clinically isolated syndrome, Prognosis, Genetic risk

## Abstract

**Introduction:**

Serum neurofilament light chain (NfL) and glial fibrillary acidic protein (GFAP) may synergistically enhance early risk stratification of multiple sclerosis (MS) diagnosis after clinically isolated syndromes (CIS). We investigated the prognostic value of combined NfL and GFAP for McDonald 2024 MS diagnosis after CIS and associations with key genetic and environmental risk factors.

**Methods:**

CIS participants, within six months after symptom onset, were included in a prospective cohort. We measured baseline serum NfL and GFAP levels and calculated z-scores. We evaluated weighted genetic risk scores for MS susceptibility, HLA-DRB1*15:01 risk and measured Anti-Epstein Barr virus Nuclear Antigen-1 (anti-EBNA1) immunoglobulin G (IgG) antibodies. Associations with MS diagnosis were evaluated using Cox proportional hazards models and time-dependent receiver operating characteristic (ROC) analyses.

**Results:**

During follow-up, 162/221 CIS participants were diagnosed with McDonald 2024 MS. Separately, high NfL and GFAP associated with earlier MS diagnoses (hazard ratio (HR) 1.36, 95% confidence interval (CI) 1.12–1.66, *p* = 0.002, HR 1.12, 95% CI 1.02–1.42, *p* = 0.01, respectively). In combined models, only NfL remained independently predictive (HR 1.30, 95% CI 1.02–1.60, *p* = 0.01). Time-dependent ROC analyses showed similar results for NfL alone and combined with GFAP. HLA-DRB1*15:01-risk, but not GFAP or anti-EBNA1 IgG, improved predictive value.

**Conclusion:**

Our study found that serum NfL outperformed GFAP in predicting early MS diagnoses after CIS. Baseline NfL, together with HLA-DRB1*15:01 status, provides robust early risk stratification for MS after CIS, whereas GFAP and anti-EBNA1 titres add limited prognostic value. Additional immunological and imaging markers are essential to further refine predictive models.

**Supplementary Information:**

The online version contains supplementary material available at 10.1007/s00415-026-13667-7.

## Introduction

Multiple sclerosis (MS) is a chronic inflammatory disease of the central nervous system (CNS) with marked heterogeneity in disease course and severity [1]. There is growing evidence that MS is often preceded by a prodromal period of subclinical disease activity [2, 3]. The first episode causing focal symptoms is referred to as clinically isolated syndrome (CIS) [3–5]. The disease course following a CIS is highly variable. Biologically, these divergent courses reflect whether CIS is due to a single inflammatory event or is rather part of a chronic compartmentalised inflammatory disease that drives the MS-associated demyelination, neuroaxonal injury, and ultimately clinical progression [6]. MS aetiology involves a complex interplay of both environmental and genetic factors [6]. Genetic susceptibility is strongly conferred by the human leukocyte antigen (HLA) DRB1*15:01 locus, in addition to more than 200 other genetic variants as discovered by genome-wide association studies (GWAS) [7, 8]. Alongside genetic predisposition, environmental exposure, particularly to Epstein–Barr virus (EBV) infection, has been consistently linked to MS risk [9]. Interactions between HLA genotype and EBV exposure may further modulate the risk of developing MS [10].

Fluid biomarkers have emerged as promising tools to improve early risk stratification [11]. Among them, neurofilament light chain (NfL) is a well-validated biomarker of neuroaxonal injury [11, 12]. Elevated CSF NfL levels are observed in both MS and CIS and are associated with early conversion to clinically definite MS (CDMS) and disability progression [13, 14]. Serum NfL closely correlates with CSF levels, reflects clinical and radiological disease activity, and independently predicts early MS diagnosis in CIS patients [15–18]. Glial fibrillary acidic protein (GFAP), another cytoskeletal protein specific to astrocytes, has been linked to disease activity and disability progression: while capturing acute inflammation to a lesser extent [19, 20], serum GFAP levels have been shown to be associated with progressive disease processes independent of relapses [21–23], correlating with EDSS progression and MRI lesion burden [22, 24, 25].

Several studies suggest a correlation between serum NfL and GFAP, with evidence indicating that the two biomarkers capture complementary aspects of MS pathology: NfL mainly reflects acute neuroaxonal damage while GFAP reflects compartmentalised inflammation [21, 22, 24, 25]. Evaluating these biomarkers together may therefore provide a more complete assessment of disease activity and may improve early prediction of MS risk. However, despite their individual prognostic value, the potential added benefit of combining them to predict MS after CIS has not been systematically investigated. Additionally, it remains unclear how these biomarkers correlate with established genetic and environmental risk factors of MS susceptibility. Addressing this knowledge gap may sharpen risk stratification of an MS diagnosis. Early risk stratification becomes increasingly important under the 2024 McDonald criteria [26], which enable earlier MS diagnosis and highlight the need to identify high-risk individuals for timely intervention. Hence, the primary aim of this study is to determine the prognostic value of combined serum NfL and GFAP for predicting an MS diagnosis during follow-up after CIS. As secondary objectives, we will examine their association with environmental and genetic risk factors.

## Methods

### Study design and participants

The study is embedded in the PROUD study, a prospective observational cohort at the Erasmus MC University Medical Centre in Rotterdam, the Netherlands. We enrolled patients with CIS, aged 18–65 years, within six months after symptom onset, and the cohort includes participants with monophasic CIS and with subsequent MS diagnosis during follow-up. The study reflects routine clinical practice with annual outpatient assessments and additional visits when clinically indicated. At baseline, participants underwent routine laboratory tests and MRIs to rule out alternative diagnoses. Individuals were excluded from analyses if a diagnosis other than CNS demyelination suggestive of MS was established. For the present study, the study period lasted from November 2006 until August 2025. The Medical Ethical Committee of the Erasmus MC Rotterdam approved the study protocol (MEC-2021-0946), and written informed consent was obtained from all participants.

### Data collection

Clinical and demographic data collected included sex, age at symptom onset, CIS topography and relapses. Lumbar puncture was performed as part of the diagnostic workup, and CSF was analysed for immunoglobulin G (IgG) index and the presence of unique oligoclonal bands (OCBs) using isoelectric focusing. Baseline brain MRI was obtained, and spinal cord MRI was performed when clinically indicated. MRIs were evaluated for high lesion load (≥ 9 T2-weighted lesions), presence of gadolinium-enhancing lesions (GELs), and unfavourable lesion localisation (i.e., infratentorial or spinal cord).

### Serology

Serum samples were collected at baseline and stored at −80 °C until analysis. Serum NfL concentrations were measured using the Siemens Healthineers chemiluminescent immunoassay (CLIA) on the Atellica IM1300^®^ platform [27]. Serum GFAP concentrations were determined with the Fujirebio CLIA on the LUMIPULSE^®^ platform [28]. Epstein–Barr virus nuclear antigen-1 (EBNA1) IgG antibodies were assessed with the LIAISON^®^ CLIA on the LIAISON^®^ XL analyser (DiaSorin, Italy), with serum samples diluted (1:20) to remain within the assay’s detection range. All analyses were conducted according to the manufacturers’ instructions.

### Genotyping and common genetic variations

Genomic DNA was extracted from baseline blood samples [29]. Genotyping was performed using the Illumina Infinium High-Throughput Screening (HTS) iSelect custom 730 K SNP BeadChip array. Genotypes were imputed to the Haplotype Reference Consortium panel. Samples and variants with a genotyping call rate < 90% were excluded. Additional filtering excluded samples with a call rate < 97.5%. A Hardy–Weinberg equilibrium threshold of 1 × 10⁻^5^ was applied, and excess heterozygosity and homozygosity were assessed. Variants with a minor allele frequency < 0.05 and samples with familial relatedness were excluded. Weighted genetic risk scores (wGRS) were calculated using PRSice-2 in R [30, 31]. The MS Susceptibility GWAS was used as the base file, incorporating allelic odds ratios and allele counts of SNPs that reached genome-wide significance (P^T^ < 5 × 10⁻^8^; *n* = 173 SNPs) [8, 32]. High-resolution (4-digit) HLA typing was performed by imputation using the HLA-TAPAS algorithm to determine HLA-DRB1*15:01 status [33].

### Definitions

CIS was defined as a first clinical episode with neurological deficits suggestive of an inflammatory demyelinating disorder of the CNS, lasting ≥ 24 h, occurring in the absence of infection [5]. MS was diagnosed according to the revised McDonald 2024 criteria, based on best available diagnostic tools in this cohort, as advanced MRI measures such as the central vein sign and paramagnetic rim sign, and optical coherence tomography were not part of the historical diagnostic assessment [26, 34].

### Statistical analysis

Descriptive statistics were used to summarise baseline characteristics. Based on data distribution, we used the *T*-test, Mann–Whitney *U* test, ANOVA or Kruskal–Wallis test for continuous data and Chi-squared test or Fisher’s exact test for categorical data. Serum NfL levels were expressed as age- and body mass index (BMI) normalised z-scores, based on Simoa (Quanterix) and Siemens Healthineers assay [35]. In NfL z-score calculation, BMI was fixed on 25 for every participant as individual BMIs were unavailable, reflecting the average value in adult Western European populations. Serum GFAP levels were expressed as age- and sex normalised z-scores, calculated based on measurement with the Fujirebio assay in a reference cohorts of healthy individuals without neurological disorders [36]. Biomarker z-scores were also categorised by median and tertiles to evaluate associations with patient characteristics. Associations between biomarker levels and environmental and genetic risk factors (i.e. HLA-DRB1*15:01 status, MS susceptibility wGRS, anti-EBNA1 IgG titres) were examined using linear regression models. Correlations were analysed with Spearman’s rho. To assess the time from CIS to McDonald 2024 MS, survival analyses were performed using stratified Kaplan–Meier curves based on biomarker cut-offs. To determine the optimal cut-off value, maximally selected rank statistics was used. Associations between NfL, GFAP and MS diagnosis were analysed using Cox proportional hazards models, adjusted for potential confounders (sex, age at onset, and high lesion load at baseline MRI). Models were constructed including covariates NfL and GFAP separately, as well as a combined model incorporating both NfL and GFAP z-scores, to analyse the added predictive value of combined biomarkers. Additional models were assessed to evaluate the contribution of genetic and environmental risk factors. Model performance was compared using concordance index (C-index) and likelihood-ratio tests (LTR). Analyses included only complete cases, with participants not reaching the endpoint censored at the end of follow-up. Results are expressed as hazard ratios (HR) with 95% confidence intervals (CI). Proportional hazards assumption was assessed using Schoenfeld residuals. To evaluate the additive value of combined biomarkers models over time, time-dependent receiver operating characteristic (ROC) curves were constructed. The area under the curve (AUC) at specific follow-up intervals was calculated to assess predictive performance, and differences in AUC between models were used to quantify the added value of biomarkers and risk factors. Statistical significance was defined as *p* < 0.05. All analyses were performed using R version 4.4.2.

## Results

### Baseline characteristics

**Fig. 1:**
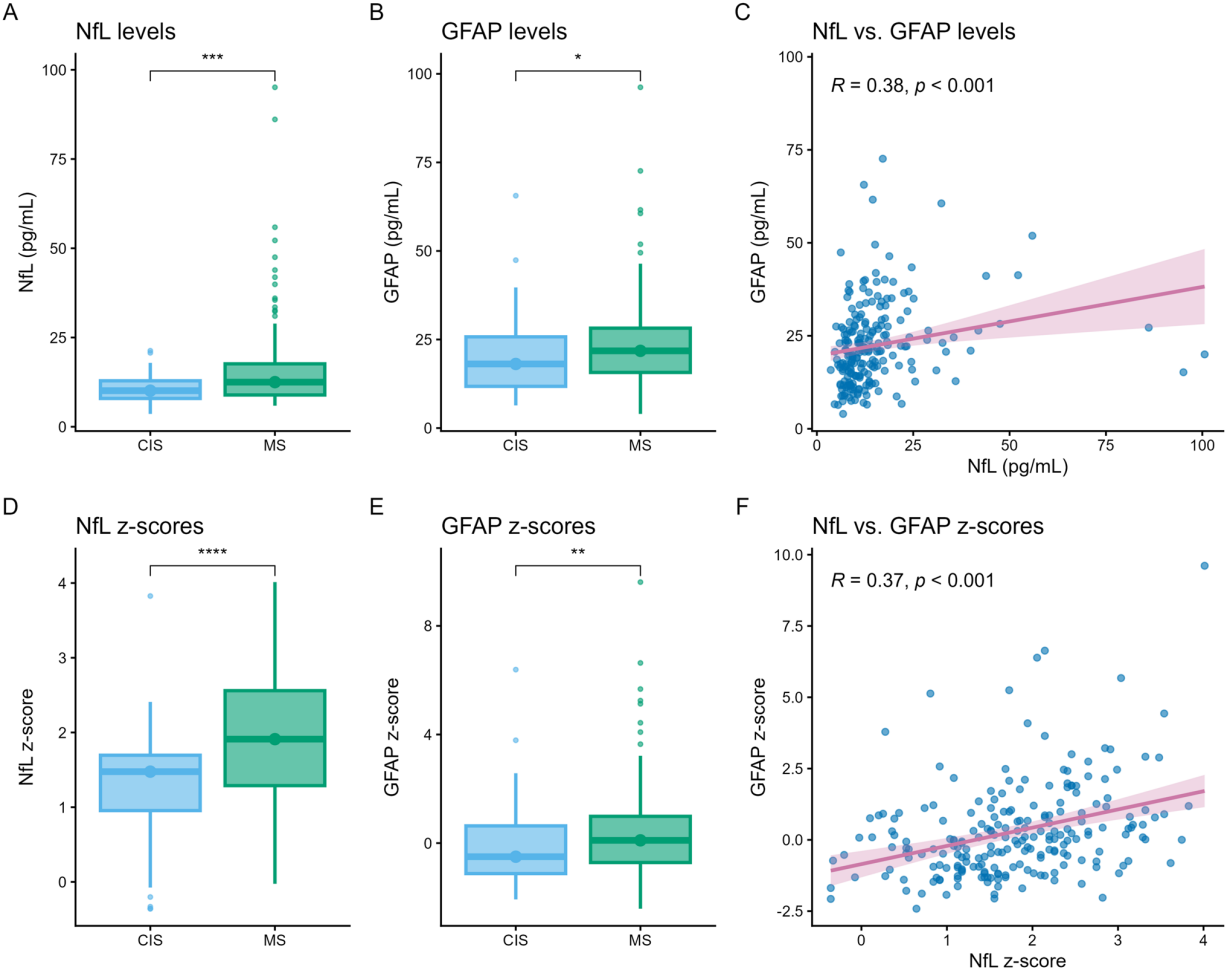
NfL and GFAP levels, z-scores, and correlations. The distribution of raw NfL (**A**) and GFAP (**B**) levels is shown, with significant differences between participants with CIS and MS. Similarly, **D** and **E** provide the z-scores for NfL (**D**) and GFAP (**E**), with significant different distributions between CIS and MS participants. Correlations between NfL and GFAP are shown for raw levels (**C**) and z-scores (**F**)

A total of 221 CIS participants were included (Supplementary Figure S1). The median age at CIS was 33.0 years (interquartile range [IQR] 27.3, 40.2), follow-up duration 5.0 years (IQR 2.7, 8.4), and time from CIS to sampling 88.0 days (IQR 43.0, 144.0) (Table 1). During follow-up, 162 (73.3%) of CIS participants were diagnosed with McDonald 2024 MS. Participants diagnosed with MS were significantly younger at the time of CIS and had longer follow-up duration, while sex, CIS topography, and time from CIS to sampling were evenly distributed. Participants with a subsequent MS diagnosis showed a higher IgG-index, were more frequently OCB-positive, and had a higher lesion load with more frequent GELs, infratentorial, and spinal cord lesions on baseline MRI (Table 1). The wGRS of MS susceptibility, HLA-DRB1*15:01, EBV seropositivity, and anti-EBNA1 IgG levels were similarly distributed between the groups (Table 1). The median serum NfL level was 11.80 pg/mL (IQR 8.60, 16.40) with a median NfL z-score of 1.71 (IQR 1.13, 2.37), and the median serum GFAP was 20.90 pg/mL (IQR 14.80, 27.50) with a median GFAP z-score of 0.00 (IQR −0.90, 0.93) (Table 1). Levels and z-scores of both NfL and GFAP were higher in participants who developed MS compared to participants who did not (Fig. 1), and showed moderate correlation (*ρ* = 0.38, *p* < 0.001 and *ρ* = 0.37, *p* < 0.001, respectively, Fig. 1).

**Table 1:** Baseline characteristics

	Total	CIS	MS	*p*-value
*N* (%)	221	59 (26.7)	162 (73.3)	
Sex (female, %)	150 (67.9)	38 (64.4)	112 (69.1)	0.61^a^
Age at CIS (years)	33.0 [27.3, 40.2]	36.8 [30.2, 43.7]	31.8 [26.7, 39.6]	0.01*^b^
Follow-up time (years)	5.0 [2.7, 8.4]	4.4 [2.3, 7.6]	5.4 [2.9, 8.7]	< 0.05*^b^
Time CIS-sampling (days)	88.0 [43.0, 144.0]	98.0 [38.5, 151.5]	84.0 [43.3, 136.8]	0.62^b^
CIS topography				
Optic nerve	96 (43.4)	24 (40.7)	72 (44.4)	0.71^a^
Brainstem	39 (17.6)	8 (13.6)	31 (19.1)	0.54^a^
Cerebellar	11 (5.0)	3 (5.1)	8 (4.9)	1.00^c^
Cerebral	23 (10.4)	3 (5.1)	20 (12.3)	0.20^c^
Spinal cord	81 (36.7)	22 (37.3)	59 (36.4)	0.89^a^
LP at baseline	165 (74.7)	41 (69.5)	124 (76.5)	
CSF IgG index	0.68 [0.55, 1.06]	0.55 [0.52, 0.66]	0.77 [0.60, 1.19]	< 0.001*^b^
CSF OCBs	113 (69.8)	11 (26.8)	102 (84.3)	< 0.001*^a^
MRI baseline				
≥ 9 T2 lesions	79 (35.7)	3 (5.1)	76 (46.9)	< 0.001*^a^
≥ 1 GEL	66 (29.9)	5 (8.5)	61 (37.7)	< 0.001*^a^
Infratentorial	78 (35.3)	9 (15.3)	69 (42.6)	< 0.001*^a^
Spinal cord	94 (42.5)	22 (37.3)	72 (44.4)	0.01*^a^
Risk factors				
wGRS MS susceptibility	7.32 (1.17)	7.17 (0.88)	7.38 (1.26)	0.18^d^
HLA-DRB1*15:01 (risk)	83 (42.3)	17 (31.5)	66 (46.5)	0.08^a^
Anti-EBNA1 IgG	591.0 [240.0, 1620.0]	518.0 [108.5, 1332.5]	648.0 [277.0, 1630.0]	0.10^a^
EBV seropositivity	199 (97.1)	52 (92.9)	147 (98.7)	0.08^c^
Biomarkers				
Serum NfL (pg/mL)	11.80 [8.60, 16.40]	10.30 [7.95, 13.30]	12.55 [8.90, 17.78]	0.001*^b^
NfL z-score	1.71 [1.13, 2.37]	1.48 [0.95, 1.70]	1.91 [1.29, 2.56]	< 0.001*^b^
Serum GFAP (pg/mL)	20.90 [14.80, 27.50]	18.10 [11.77, 25.75]	21.80 [15.70, 28.20]	0.02*^b^
GFAP z-score	0.00 [−0.90, 0.93]	−0.50 [−1.12, 0.63]	0.10 [−0.71, 0.99]	0.01*^b^

Values are represented in n (%), median with interquartile range [IQR] or mean with standard deviation (SD)

*CIS* clinically isolated syndrome, *CSF* cerebrospinal fluid, *GEL* gadolinium-enhancing lesion, *EBNA1* Epstein Barr virus nuclear antigen-1, *EBV* Epstein Barr virus, *GFAP* glial fibrillary acidic protein, *HLA* human leukocyte antigen, *IgG* immunoglobulin G, *LP* lumbar puncture, *MS* multiple sclerosis, *NfL* neurofilament light chain, *OCBs* oligoclonal bands, *wGRS* weighted genetic risk score

*Statistically significant with *p*-value < 0.05

a = Chi-squared test; b = Mann–Whitney *U* test; c = Fisher’s exact test, d = Student’s *T*-test

### Correlation of serum NfL and GFAP with baseline characteristics

Baseline characteristics by NfL and GFAP z-score tertiles are shown in Supplementary Table S1 and S2. Participants in the highest NfL z-score tertile were younger at CIS and presented more often with brainstem symptoms. The highest NfL tertile was associated with high lesion load, GELs, and infratentorial lesions at baseline MRI. For GFAP z-score tertiles, the highest tertile showed more GELs and spinal cord lesions at baseline MRI, with no other differences (Supplementary Table S2).

### Serum NfL, GFAP and early disease course

**Fig. 2:**
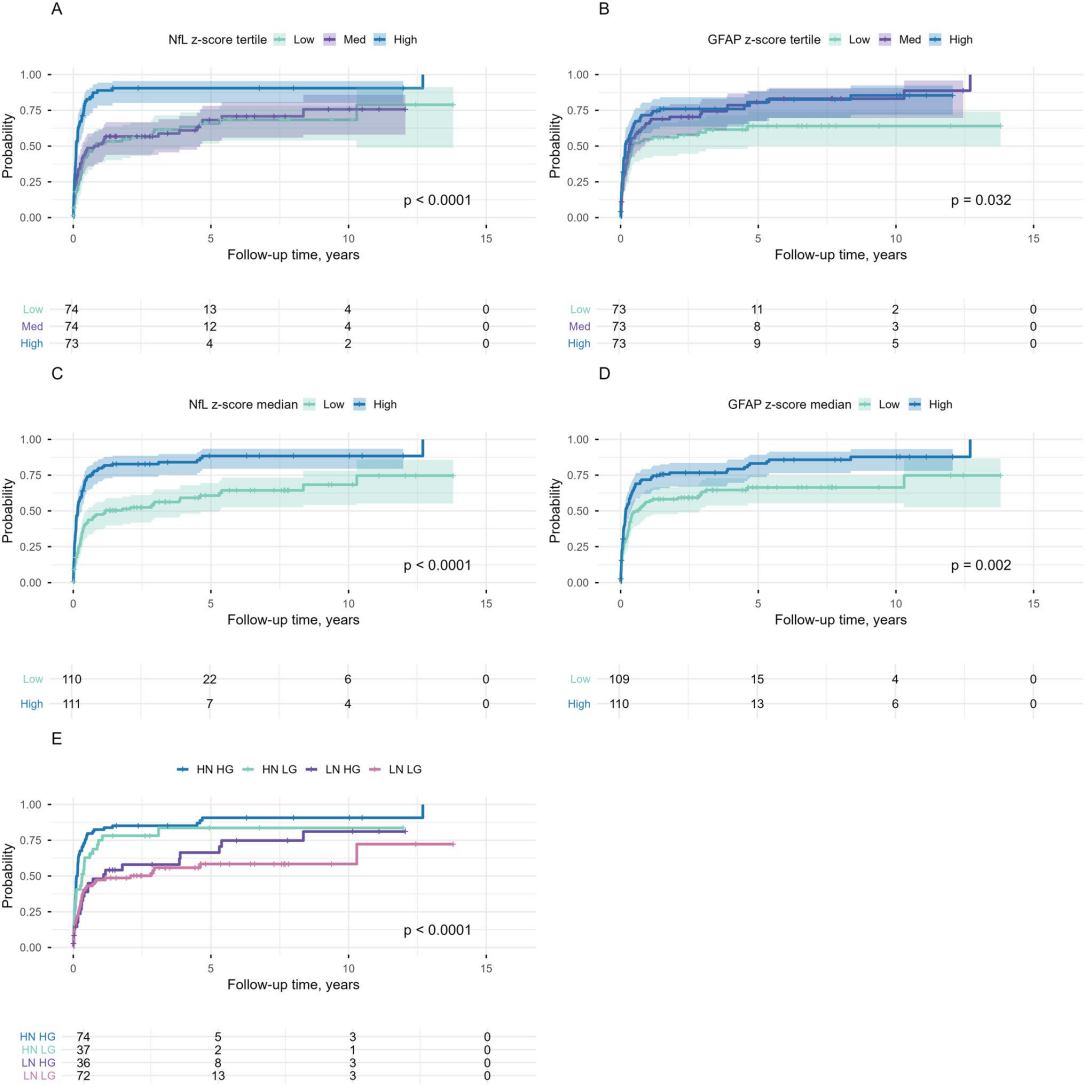
Kaplan–Meier curves for time to McDonald 2024 MS diagnosis. **A** shows the survival analyses of time to McDonald 2024 MS diagnosis by tertiles of NfL, with significant differences between High > 2.20 (blue), Medium 1.41–2.20 (purple) and Low ≤ 1.41 (green). In **B**, time to MS diagnosis is shown for GFAP tertiles, with no significant differences by tertile subgroups (High > 0.53 [blue], Medium −0.53–0.53 [purple], Low ≤ −0.53 [green]). Panel **C** and **D** show the time to MS diagnosis, with NfL (**C**) divided by median into High ≥ 1.71 (blue) and Low < 1.71 (green), and for GFAP (**D**) High ≥ 0.00 (blue) and Low < 0.00 (green), with significant differences in both analyses between High and Low. **E** shows a composite stratification of NfL and GFAP, with faster MS diagnoses in High NfL & High GFAP (blue) and High NfL & Low GFAP (green), compared to Low NfL & High GFAP (purple) and Low NfL & Low GFAP (pink)

**Fig. 3:**
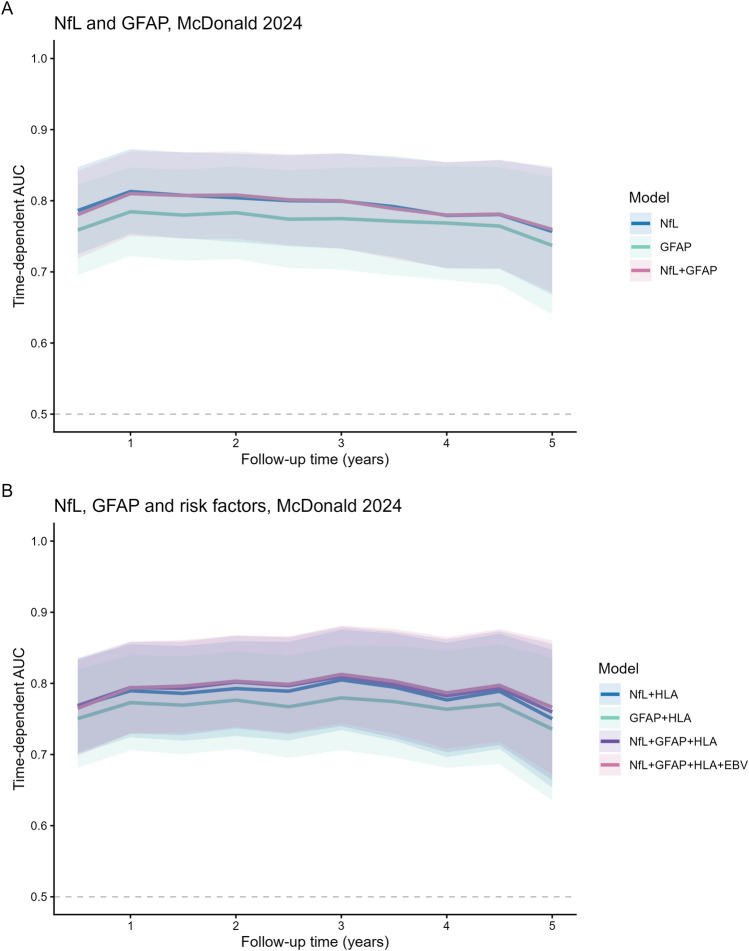
Time-dependent ROC curve for NfL and GFAP in the first 5 years of follow-up. In **A**, the time-dependent ROC curves for NfL, GFAP, and combined NfL + GFAP are shown, with similar curves for NfL and NfL + GFAP compared to GFAP alone. In **B**, HLA-DRB1*15:01 and anti-EBNA1 IgG levels were added as other known mediators for MS susceptibility, with similar curves for NfL with HLA-DRB1*15:01, as combined with GFAP and as a complete model with NfL + GFAP and both HLA-DRB1*15:01 and anti-EBNA1 IgG levels, in comparison to GFAP + HLA alone

Cumulative probability of a McDonald 2024 MS diagnosis was evaluated with Kaplan–Meier survival analysis (Fig. 2). For NfL, median time to MS diagnosis differed significantly between when NfL z-scores were divided into tertiles (High 0.11, Medium 0.83, Low 0.77 years, log-rank *p* < 0.001, Fig. 2A) and if dichotomised (High 0.16, Low 1.17 years, *p* < 0.001, Fig. 2C). For GFAP, analysis by z-score tertiles showed significant differences in median time to MS diagnosis (High 0.17, Medium 0.33, Low 0.44 log-rank *p* = 0.03, Fig. 2B), and when dichotomised on median (High 0.18, Low 0.60 years, log-rank *p* = 0.002, Fig. 2D). When these biomarkers were combined based on their dichotomised z-scores (Fig. 2E), median time to MS diagnosis was shorter for participants with High NfL & High GFAP and High NfL & Low GFAP compared to Low NfL & High GFAP and Low NfL & Low GFAP (respectively 0.14, 0.32, 1.10 and 2.08 years, log-rank *p* < 0.001). In pairwise comparisons, significant differences were observed only between High NfL & High GFAP vs. Low NfL & High GFAP (*p* = 0.003), High NfL & High GFAP vs. Low NfL & Low GFAP (*p* < 0.001) and High NfL & Low GFAP vs. Low NfL & Low GFAP (*p* = 0.01), while other comparisons did not differ. In Supplementary Figure S2, zoomed-in views of the first 5 years of the Kaplan–Meier plots are provided to facilitate visualisation of early divergence between groups. In separate Cox proportional hazard models (Table 2), NfL and GFAP z-scores were both associated with shorter time to MS diagnosis (NfL HR 1.36 (1.12–1.66), *p* = 0.002 and GFAP (HR 1.12, 95% CI 1.02–1.42, *p* = 0.01). When combining both NfL and GFAP, NfL remained independently predictive (HR 1.30, 95% CI 1.05–1.60, *p* = 0.01), while GFAP did not (HR 1.07, 95% CI 0.97–1.18, *p* = 0.15). Comparing the combined model NfL + GFAP with NfL alone did not show a significant improvement (LRT *χ*^2^ 1.96, *p* = 0.16), indicating that adding GFAP did not increase prognostic accuracy. Additionally, time-dependent ROC analyses showed that AUCs of the combined model NfL + GFAP were similar to those of individual NfL (Fig. 3). The incremental discriminative value of adding NfL and GFAP together was negligible at year 1, 3, and 5 (Table 3).

**Table 2:** Cox regression analyses for time to McDonald 2024 MS of NfL and GFAP

Model	Variable	HR (95% CI)	*p*-value	C-index (SE)	LRT *χ*^2^ (*p*-value)
NfL and GFAP					
NfL	NfL z-score	1.36 (1.12–1.66)	0.002*	0.695 (0.02)	
GFAP	GFAP z-score	1.12 (1.02–1.42)	0.01*	0.680 (0.02)	
NfL + GFAP	NfL z-score	1.30 (1.05–1.60)	0.01*	0.693 (0.02)	1.96 (0.16)^a^
	GFAP z-score	1.07 (0.97–1.18)	0.15		
NfL, GFAP and risk factors					
NfL + HLA	NfL z-score	1.33 (1.01–1.67)	0.01*	0.692 (0.02)	4.28 (0.04*)^b^
	HLA-DRB1*15:01	1.45 (1.01–2.01)	0.04*		
GFAP + HLA	GFAP z-score	1.11 (1.01–1.22)	0.04*	0.683 (0.03)	3.70 (0.05)^c^
	HLA-DRB1*15:01	1.41 (0.99–2.02)	0.05		
NfL + GFAP + HLA	NfL z-score	1.27 (1.00–1.60	< 0.05*	0.694 (0.02)	1.33 (0.25)^d^
	GFAP z-score	1.07 (0.96–1.18)	0.24		
	HLA-DRB1*15:01	1.31 (0.99–2.01)	0.05		
NfL + GFAP + HLA + EBV	NfL z-score	1.27 (1.00–1.60)	< 0.05*	0.692 (0.02)	2.01 (0.36)^e^
	GFAP z-score	1.07 (0.96–1.19)	0.21		
	HLA-DRB1*15:01	1.37 (0.96–1.96)	0.09		
	Anti-EBNA1 IgG	1.05 (0.94–1.17)	0.40		

Analyses were performed with multivariate Cox proportional hazards models, adjusted for sex, age and ≥ 9 T2 lesions on baseline MRI. Anti-EBNA1 IgG titres are log-transformed. Only complete cases were used, with population in analyses of NfL + GFAP *n* = 217 and analyses of NfL + GFAP + risk factors *n* = 189

Additive value of combined biomarkers was evaluated using Likelihood Ratio Test (LTR). Model comparisons: a = NfL vs. NfL + GFAP, b = NfL vs. NfL + HLA, c = GFAP vs. GFAP + HLA, d = NfL + HLA vs. NfL + GFAP + HLA and e = NfL + HLA vs. NfL + GFAP + HLA + EBV

*CI* confidence interval, *CIS* clinically isolated syndrome, *EBNA1* Epstein Barr virus nuclear antigen-1, *EBV* Epstein Barr virus, *GFAP* glial fibrillary acidic protein, *HLA* human leukocyte antigen, *HR* hazard ratio, *IgG* immunoglobulin G, *LRT* likelihood ratio test, *NfL* neurofilament light chain, *SE* standard error

*Statistically significant with *p*-value < 0.05

**Table 3:** Time-dependent ROC analyses for prediction McDonald 2024 MS diagnosis after CIS

	AUC NfL (95% CI)	AUC GFAP (95% CI)	AUC NfL + GFAP (95% CI)	ΔAUC^a^
NfL and GFAP				
Year 1	0.81	0.78	0.81	−0.0029
Year 3	0.80	0.77	0.80	0.0005
Year 5	0.76	0.74	0.76	0.0028

	AUC NfL + HLA (95% CI)	AUC GFAP + HLA (95% CI)	AUC NfL + GFAP + HLA (95% CI)	AUC NfL + GFAP + HLA + EBV (95% CI)	ΔAUC^b^	ΔAUC^c^	ΔAUC^d^
NfL, GFAP and risk factors							
Year 1	0.80	0.77	0.79	0.79	0.0045	0.0042	−0.0003
Year 3	0.80	0.78	0.81	0.81	0.0047	0.0077	0.0030
Year 5	0.75	0.74	0.76	0.77	0.0096	0.0162	0.0066

Analyses were performed with time-dependent receiver operating characteristic (ROC) analysis for prediction of MS diagnosis after CIS, based on Cox proportional hazards model, adjusted for sex, age and ≥ 9 T2 lesions on baseline MRI. Time point indicates years after CIS. At 5 years, 13.4% (*n* = 29) was still at risk for an MS diagnosis. ΔAUC represents the difference in AUC between models, with a = NfL vs. NfL + GFAP, b = NfL + HLA vs. NfL + GFAP + HLA, c = NfL + HLA vs. NfL + GFAP + HLA + EBV, d = NfL + GFAP + HLA vs. NfL + GFAP + HLA + EBNA1

*AUC* area under the curve, *CI* confidence interval, *CIS* clinically isolated syndrome, *EBNA1* Epstein Barr virus nuclear antigen-1, *EBV* Epstein Barr virus, *GFAP* glial fibrillary acidic protein, *HLA* human leukocyte antigen, *NfL* neurofilament light chain, *ROC* receiver operating characteristic, *SE* standard error

Since NfL showed the best prognostic value, we identified the optimal cut-off value for NfL z-score at 2.26, using maximally selected rank statistics that stratified participants by survival (Supplementary Figure S3). Sensitivity analyses comparing participants with samples collected within versus after 3 months showed no differences in the results (data not shown). Additional sensitivity analyses explored additional value of NfL beyond baseline MRI measures with Cox proportional hazards models. In models without serum NfL, both high T2 lesion load and the presence of GELs were independently associated with early MS diagnosis (Supplementary Table S3). When serum NfL was added to the model, NfL, high T2 lesion load and the presence of GELs remained significant predictors, and likelihood-ratio testing indicated that inclusion of NfL provided additional explanatory value to the MRI variables.

### Serum NfL, GFAP and genetic and environmental risk factors

**Fig. 4:**
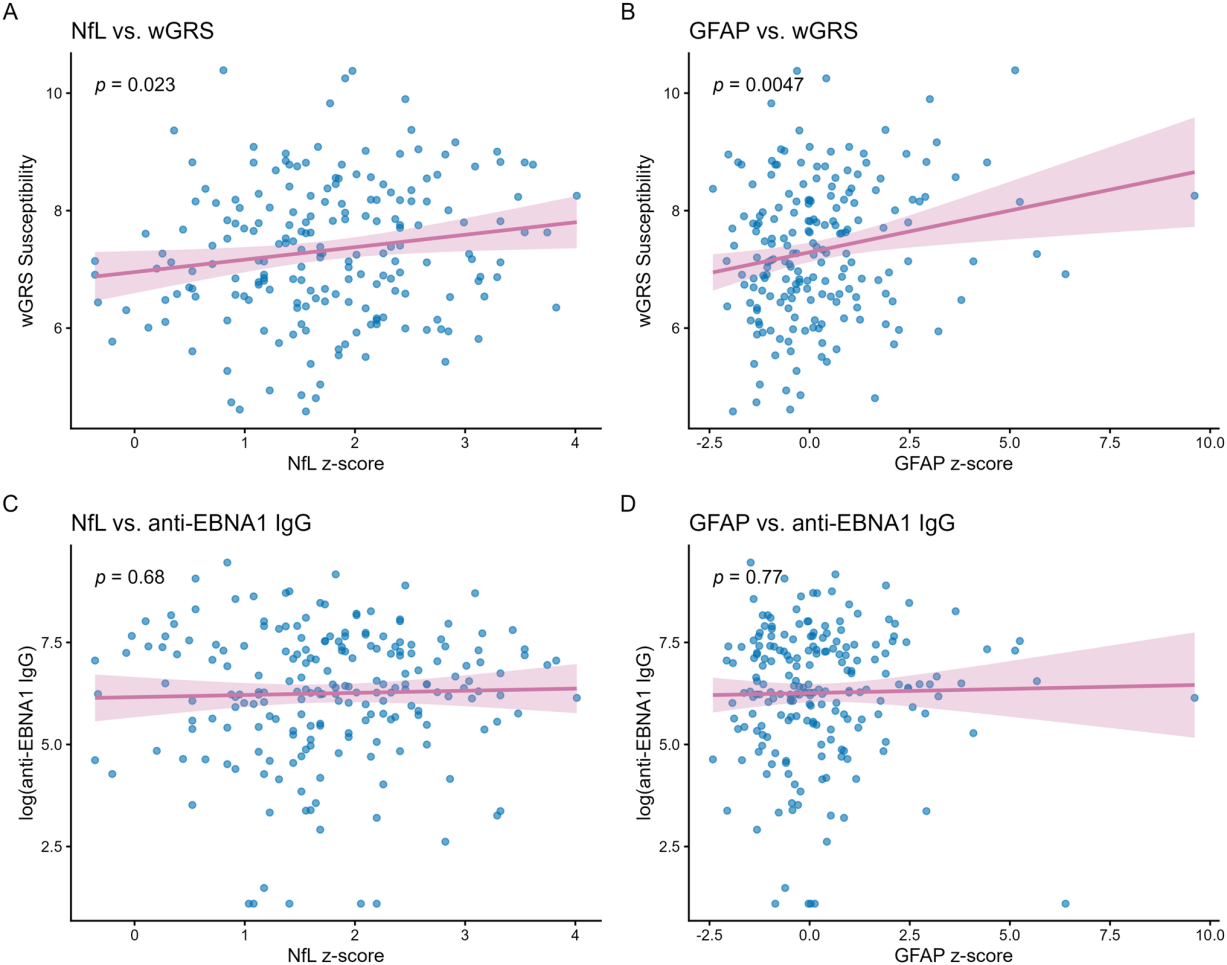
Associations of NfL and GFAP with genetic and environmental risk factors. The upper row shows the association of NfL (**A**) and GFAP (**B**) with MS susceptibility wGRS. In the lower row, no association of NfL (**C**) and GFAP (**D**) with anti-EBNA1 IgG levels is found

To further explore the prognostic value of biomarkers, we evaluated associations with established genetic and environmental risk factors. In univariate linear regression, MS susceptibility wGRS was associated with both NfL (*β* = 0.12, 95% CI 0.02–0.23, *p* = 0.02) and GFAP z-scores (*β* = 0.29, 95% CI 0.09–0.49, *p* = 0.005). We found no association of HLA-DRB1*15:01 with NfL (*β* = 0.20, 95% CI −0.05–0.46, *p* = 0.12), but it associated with GFAP (*β* = 0.52, 95% CI 0.04–0.99, *p* = 0.03). Anti-EBNA1 IgG did not associate with NfL or GFAP (respectively *β* = 0.02, 95% CI −0.06–0.09, *p* = 0.68 and *β* = 0.02, 95% CI −0.12–0.16, *p* = 0.77, Fig. 4). We generated multivariate Cox models, using HLA-DRB1*15:01 and anti-EBNA1 IgG as additional covariates (Table 2, Supplementary Table S4). HLA-DRB1*15:01 was chosen above wGRS of MS susceptibility as a genetic covariate by providing a better model performance. In the model combining NfL with HLA-DRB1*15:01, these were both independently associated with shorter time to MS diagnosis (NfL HR 1.33, 95% CI 1.01–1.67, *p* = 0.01 and HLA HR 1.45, 95% CI 1.01–2.01, *p* = 0.04). Adding HLA-DRB1*15:01 to the NfL model improved performance (LRT *χ*^2^ 4.28, *p* = 0.04). Also, in the model of GFAP with HLA-DRB1*15:01, GFAP remained significantly associated with an earlier MS diagnosis (HR 1.11, 95% CI 1.01–1.22, *p* = 0.04), but HLA-DRB1*15:01 attenuated (HR 1.41, 95% CI 0.99–2.02, *p* = 0.05). When combining NfL and HLA-DRB1*15:01 with GFAP, high NfL remained independently associated with shorter time to MS diagnosis, whereas GFAP and HLA-DRB1*15:01 were no longer significantly associated, with no improvement in the model’s performance compared to NfL and HLA alone (LRT *χ*^2^ 1.33, *p* = 0.25). In the model with NfL, GFAP, HLA-DRB1*15:01, and anti-EBNA1 IgG, only NfL remained as an independent predictor. Adding GFAP and anti-EBNA1 IgG to the model of NfL and HLA-DRB1*15:01 did not show a significant improvement in model fit (Table 2, Supplementary Table S4). Time-dependent ROC analyses further indicated that including both NfL and GFAP in combination with HLA risk allele or with HLA risk allele and anti-EBNA1 IgG yielded AUCs similar to the model with NfL and HLA risk allele only (Fig. 3). The additional contribution of combining biomarkers with all risk factors was minimal across follow-up at years 1, 3, and 5 (Table 3).

## Discussion

In this prospective study of patients with CIS, we found that serum NfL outperformed serum GFAP in predicting an early McDonald 2024 MS diagnosis. Separately, increased NfL and GFAP are both associated with an MS diagnosis, but when combining both biomarkers, only the association of NfL persisted independently. After accounting for established genetic and environmental risk factors, both separate NfL and GFAP continued to be robust predictors, while HLA-DRB1*15:01 further refined risk stratification. Combining both biomarkers did not significantly improve prognostic performance for MS diagnosis compared to NfL alone. These findings highlight the potential of NfL, alone or combined with HLA-DRB1*15:01, to improve early risk prediction in CIS patients.

We demonstrated that baseline serum NfL levels were significantly higher in CIS patients with subsequent McDonald 2024 MS diagnosis compared with those who remained CIS. Elevated NfL was also associated with markers of acute inflammation, including high lesion load and gadolinium-enhancement on baseline MRI, in concordance with prior studies [16, 17]. MS patients were significantly younger at CIS presentation compared to patients who remained CIS. Likewise, the participants in the highest NfL tertile were also younger at CIS. The higher serum NfL levels observed in younger patients may reflect a more robust acute inflammatory response or greater axonal vulnerability, consistent with more inflammatory-active disease at early ages [37, 38]. While GFAP values were also elevated in participants who developed MS compared to CIS, other inflammatory characteristics were less pronounced for GFAP in relation to NfL. The absence of added value of GFAP in the combined analyses for predicting an MS diagnosis might be explained by similar reasons. Although in separate analyses both NfL and GFAP were significantly associated with an MS diagnosis, combining both biomarkers did not yield better prognostic value. In addition, NfL alone achieved similar predictive performance as combined NfL and GFAP. These observations indicate that the prognostic value of NfL to predict early inflammatory activity outweighs that of GFAP and that the incremental prognostic value of GFAP is negligible. Our findings show that serum NfL adds prognostic information on top of established MRI markers, indicating that NfL reflects complementary processes of underlying disease biology not fully reflected by radiological activity. The added value of NfL beyond MRI supports its potential role in early risk stratification and may be relevant when MRI findings are equivocal or when MRI access is complicated. While the optimal NfL z-score cut-off of 2.26 underlines the prognostic potential, the threshold may be cohort-specific and requires external validation before clinical implementation. Notably, this study incorporated the revised 2024 McDonald MS criteria, which enable earlier diagnosis and increase the importance of early risk stratification [26]. While NfL and GFAP performed well under the previous criteria [17, 18, 20], our findings highlight that these remain relevant in the 2024 framework. Altogether, our findings may indicate overlapping biological information by the two markers, with NfL as a sensitive indicator of acute inflammatory activity, directly reflecting the pathophysiological processes that drive early MS diagnosis after CIS, while GFAP may better reflect disease progression [16, 17, 21, 22, 25]. In this context, it can also be explained that combined CSF NfL and GFAP do predict long-term clinical worsening in MS, as recent evidence demonstrated that integrating both biomarkers enhances the prediction of sustained disability progression compared to using either marker alone [39].

Importantly, as elevated NfL reflects neuroaxonal damage, it is not specific to MS and may be influenced by other neurological conditions or comorbidities [40]. In addition to age, factors such as BMI, blood volume, creatinine, and glycosylated haemoglobin (HbA1c) may affect serum NfL levels [41, 42]. Evidence on confounders for serum GFAP is more limited; while it appears age-dependent, sex has also been reported to influence GFAP concentrations, with higher levels observed in women [36, 43]. Although we used confounder adjusted z-scores to account for these variations, unmeasured confounders may still have contributed to variability in biomarker levels and should be considered when interpreting our findings. Importantly, the relatively low z-scores for GFAP could result from assay-specific characteristics with scarce reference data and demographic differences in the cohort [36, 44]. Since the raw NfL levels associated also more clearly with MS-status compared to GFAP, this makes inaccuracies in z-score estimation as the sole driver of this observed difference less likely. Moreover, biologically, the lower GFAP levels may reflect the limited astrocytic injury in early CIS. These factors could contribute to the weaker associations observed for this biomarker.

Our findings suggest that genetic risk factors may differentially relate to biomarker expression and disease prognosis. Higher wGRS was associated with elevated NfL and GFAP, indicating that genetic susceptibility may predispose individuals to both axonal damage and astrocytic activation already at the CIS stage. However, this did not translate into predictive value for an earlier MS diagnosis. Only HLA-DRB1*15:01 predicted conversion to MS in survival analyses, whereas wGRS did not. This divergence may reflect the dominant effect of HLA on MS risk, while the predictive value of wGRS as a whole might be more modest [32, 45]. Together, these results suggest that genetic burden may capture biological vulnerability, and the presence of HLA-DRB1 risk allele exerts a greater impact on the clinical susceptibility to MS. Recent data from an optic neuritis study demonstrated that incorporating an MS genetic risk score can increase the predicted conversion risk from approximately 4% to over 40% [46]. From a risk stratification perspective, this highlights the importance of integrating biomarkers with genetic susceptibility in such predictive models. Beyond its independent effect, HLA status may further interact with environmental exposure, most prominently EBV infection, to drive MS risk [47]. Exploring such interactions in the context of biomarker profiles could provide further insight into how genetic and environmental factors jointly shape disease onset. Although EBNA1 serology has been firmly implicated in MS susceptibility [9], we did not observe an association of anti-EBNA1 IgG levels with baseline biomarkers levels nor a prognostic effect in CIS. This may reflect the high prevalence of EBV seropositivity in our CIS population, limiting the discriminative value at this disease stage. Alternatively, EBV may primarily drive early immune activation rather than the extent of acute neuroaxonal or astrocytic injury as captured by these biomarkers.

A strength of this study is the integrative approach, as we simultaneously incorporated serum NfL and GFAP, genetic susceptibility, and EBNA1 serostatus into a prognostic framework. This study has some limitations. First, the historical nature of the cohort, with recruitment beginning in 2006, predates the availability of contemporary MRI techniques (e.g., 3 T scanners, high-resolution 3D FLAIR), advanced lesion-characterisation methods, and optical coherence tomography. As a result, clinically relevant radiological or retinal biomarkers may have been missed, and some participants classified as CIS may have met diagnostic criteria for MS if evaluated using current imaging modalities and the 2024 McDonald criteria. These technological differences should be considered when interpreting the diagnostic distribution and the relevance of our findings to present-day clinical practice. Second, follow-up duration differed significantly between participants who did and did not receive an MS diagnosis, which is a logical consequence, as longer follow-up provides more opportunity for conversion. Yet, we accounted for these effects by using survival analyses and Cox proportional hazards models, which appropriately incorporate varying observation times. Nonetheless, estimates beyond approximately 10 years should be interpreted carefully due to the small number of participants remaining at risk during later follow-up. Third, the interval between CIS and baseline sampling varied across the cohort, ranging from a few days up to 6 months, which may affect whether biomarkers levels accurately reflect acute inflammatory activity or if this effect diminishes over time. However, this interval did not differ significantly between participants with a subsequent MS diagnosis and those without, supporting comparability of baseline measurements across groups. Additionally, sensitivity analyses stratifying samples collected within or after 3 months did not yield different results. A further limitation is that only baseline biomarker measurements were considered, limiting to assess temporal dynamics. Although longitudinal analyses may provide additional insight into disease progression, using baseline samples ensured a standardised time point at CIS for all participants, facilitating comparability across the cohort. Future work could explore longitudinal changes to improve the prediction of disease courses.

In conclusion, baseline serum NfL proved to be a robust predictor of early MS after CIS, outperforming GFAP, while HLA-DRB1*15:01 further refined risk stratification. These findings highlight the potential of integrating fluid biomarkers with key genetic markers to improve early risk prediction and guide personalised monitoring strategies. Future studies with longitudinal biomarker sampling and inclusion of additional immunological and imaging markers are essential to further refine predictive models and elucidate how these factors jointly shape early MS pathophysiology.

## Supplementary Information

Below is the link to the electronic supplementary material.

Supplementary file1 (PDF 517 KB)

## Data Availability

The data that support the findings of this study are available from the corresponding author, upon reasonable request.

## Abbreviations

AUC: Area under the curve
CIS: Clinically isolated syndrome
CNS: Central nervous system
CSF: Cerebrospinal fluid
DMTs: Disease-modifying therapies
EBNA1: Epstein–Barr nuclear antigen-1
EBV: Epstein–Barr virus
GELs: Gadolinium-enhancing lesions
GFAP: Glial fibrillary acidic protein
GWAS: Genome-wide association studies
HLA: Human leukocyte antigen
IgG: Immunoglobulin G
IMSGC: International multiple sclerosis genetics consortium
IQR: Interquartile range
LP: Lumbar puncture
MS: Multiple sclerosis
MRI: Magnetic resonance imaging
NfL: Neurofilament light chain
OCBs: Oligoclonal bands
ROC: Receiver operating characteristic
SD: Standard deviation
SE: Standard error
SNP: Single nucleotide polymorphism
wGRS: Weighted genetic risk score
