# Supplementary material for: Serum neurofilament light chain and glial fibrillary acidic protein predicting multiple sclerosis after clinically isolated syndrome
Source: J Neurol. 2026 Feb 9;273(2):128. doi: 10.1007/s00415-026-13667-7 (PMC12886267; doi:10.1007/s00415-026-13667-7)
Supplement: Supplementary file 1 — Supplementary file1 (PDF 517 KB) [file 415_2026_13667_MOESM1_ESM.pdf]

Supplementary Material

Serum neurofilament light chain and glial fibrillary acidic protein predicting multiple sclerosis after clinically isolated syndrome

Authors:
Cato E.A. Corsten[1], Veerle S.A. Geraedts[1], Ana M. Marques[2], Marie-José Melief[2], Barry Koelewijn – van Vliet[3], Jeroen van Rooij[4], Marcello Ciaccio[5], Luisa Agnello[5], Jens Kuhle[6], Andrei N. Tintu[3], Beatrijs Wokke[1], and Joost Smolders [1,2,7]

1. Department of Neurology, MS Center ErasMS, Erasmus MC University Medical Center, Rotterdam, the Netherlands
2. Department of Immunology, MS Center ErasMS, Erasmus MC University Medical Center, Rotterdam, the Netherlands
3. Department of Clinical Chemistry, Erasmus MC University Medical Center, Rotterdam, the Netherlands.
4. Department of Internal Medicine, Erasmus MC University Medical Center, Rotterdam, the Netherlands
5. Department of Biomedicine, Neurosciences and Advanced Diagnostics, Institute of Clinical Biochemistry, Clinical Molecular Medicine and Clinical Laboratory Medicine, University of Palermo, Palermo, Italy
6. Multiple Sclerosis Centre, Neurology, Departments of Clinical Research and Biomedicine, Research Centre for Clinical Neuroimmunology and Neuroscience, University of Basel and University Hospital Basel, Basel, Switzerland
7. Neuroimmunology Research Group, Netherlands Institute for Neuroscience, Amsterdam, the Netherlands

Corresponding author:
Dr. Joost Smolders, MS Center ErasMS, Department of Neurology, Erasmus MC University Medical Center, Dr. Molewaterplein 40, 3015 GD Rotterdam, The Netherlands.
E-mail: j.j.f.m.smolders@erasmusmc.nl

Table of content:

- Figure S1 Flowchart of participant and sample selection
- Table S1 Clinical and demographic characteristics by NfL z-score tertiles
- Table S2 Clinical and demographic characteristics by GFAP z-score tertiles
- Figure S2 Zoomed-in Kaplan-Meier curves for time to McDonald 2024 MS diagnosis
- Figure S3 Maximally selected rank statistics of NfL
- Table S3 Sensitivity analyses of Cox regression analyses of MRI and NfL
- Table S4 Additional results of Cox regression analyses of NfL, GFAP and risk factors

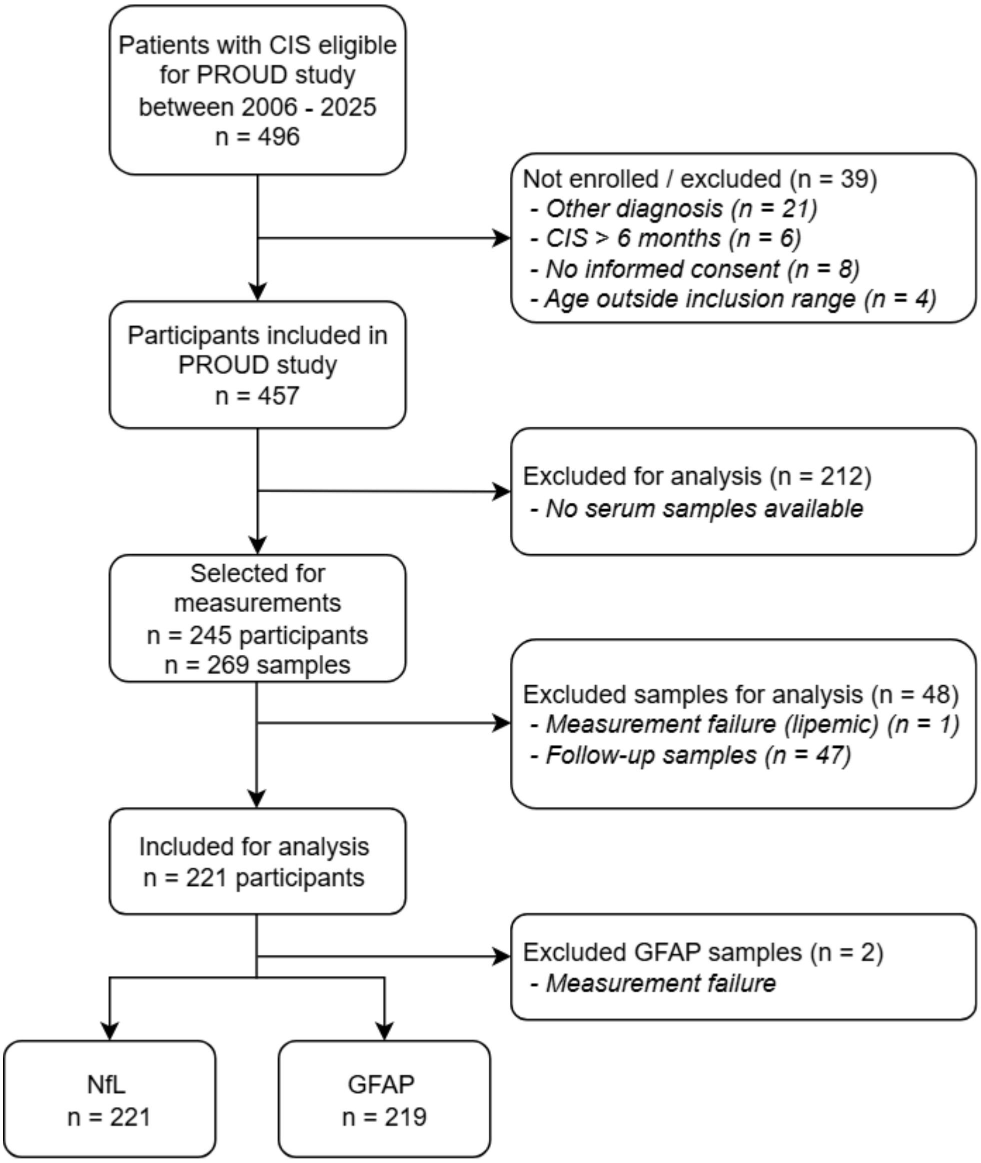

Figure S1 Flowchart of participant and sample selection

Table S1 | Clinical and demographic characteristics by NfL z-score tertiles

	**Low (≤1.41)**	**Medium (1.41–2.20)**	**High (>2.20)**	**p-value**
N	74	74	73	
Sex (female, %)	49 (66.2)	50 (67.6)	51 (69.9)	0.89 [b]
Age at CIS (years)	34.4 [29.5, 42.5]	38.0 [29.4, 43.1]	30.0 [24.1, 35.3]	<0.001* [a]
Follow-up time (years)	5.1 [2.9, 7.8]	4.9 [2.6, 9.3]	5.3 [2.7, 8.0]	0.91 [a]
Time CIS-sampling (days)	96.0 [48.0, 153.0]	90.0 [25.8, 142.8]	78.0 [49.0, 127.0]	0.37 [a]
CIS topography				
Optic nerve	30 (40.5)	41 (55.4)	25 (34.2)	0.05 [b]
Brainstem	11 (14.9)	8 (10.8)	20 (27.4)	0.03* [b]
Cerebellar	3 (4.1)	1 (1.4)	7 (9.6)	0.07 [c]
Cerebral	7 (9.5)	6 (8.1)	10 (13.7)	0.53 [c]
Spinal cord	27 (36.5)	26 (35.1)	28 (38.4)	0.82 [b]
LP at baseline	58 (78.4)	54 (73.0)	53 (72.6)	
CSF IgG index	0.62 [0.54, 0.88]	0.63 [0.56, 0.92]	0.83 [0.60, 1.19]	0.08 [a]
CSF OCBs	38 (65.5)	35 (66.0)	40 (78.4)	0.27 [b]
MRI baseline				
≥9 T2 lesions	17 (23.0)	21 (28.4)	41 (56.2)	<0.001* [b]
≥1 GEL	11 (14.9)	21 (28.4)	34 (46.6)	<0.001* [b]
Infratentorial	19 (25.7)	19 (25.7)	40 (54.8)	<0.001* [b]
Spinal cord	30 (40.5)	29 (39.2)	35 (47.9)	0.18 [b]

Values are represented in n, % or median with interquartile range [IQR].
Abbreviations: CIS: clinically isolated syndrome. CSF: cerebrospinal fluid. GEL: gadolinium-enhancing lesion. IgG: immunoglobulin G. IQR: interquartile range. LP: lumbar puncture. MS: multiple sclerosis. NfL: neurofilament light chain. OCBs: oligoclonal bands.
* statistically significant with p-value <0.05
a = Kruskal-Wallis, b = Chi-squared test; c = Fisher's exact

Table S2 | Clinical and demographic characteristics by GFAP z-score tertiles				
	**Low (≤-0.53)**	**Medium (-0.53–0.53)**	**High (>0.53)**	**p-value**
N	73	73	73	
Sex (female, %)	54 (74.0)	49 (67.1)	46 (63.0)	0.36 [b]
Age at CIS (years)	32.3 [27.7, 41.8]	31.2 [24.7, 40.1]	33.6 [28.6, 39.6]	0.44 [a]
Follow-up time (years)	4.8 [2.9, 7.6]	5.0 [2.5, 7.7]	5.4 [2.6, 9.3]	0.78 [a]
Time CIS-sampling (days)	96.0 [38.0, 153.0]	90.0 [36.0, 142.0]	82.0 [49.0, 132.0]	0.85 [a]
CIS topography				
Optic nerve	35 (47.9)	29 (39.7)	30 (41.1)	0.36 b
Brainstem	10 (13.7)	10 (13.7)	18 (24.7)	0.11 [b]
Cerebellar	2 (2.7)	3 (4.1)	6 (8.2)	0.26 [c]
Cerebral	7 (9.6)	5 (6.8)	11 (15.1)	0.28 [c]
Spinal cord	23 (31.5)	30 (41.1)	28 (38.4)	0.38 [b]
LP at baseline	64 (87.7)	52 (71.2)	47 (64.4)	
CSF IgG index	0.63 [0.54, 1.18]	0.69 [0.56, 0.84]	0.69 [0.57, 1.11]	0.63 [a]
CSF OCBs	38 (60.3)	38 (76.0)	36 (76.6)	0.09 [b]
MRI baseline				
≥9 T2 lesions	23 (31.5)	26 (35.6)	30 (41.1)	0.44 [b]
≥1 GEL	13 (17.8)	24 (32.9)	29 (39.7)	0.01* [b]
Infratentorial	20 (27.4)	30 (41.1)	27 (37.0)	0.18 [b]
Spinal cord	23 (31.5)	34 (46.6)	36 (49.3)	0.002* [b]

Values are represented in n, % or median with interquartile range [IQR].
Abbreviations: CIS: clinically isolated syndrome. CSF: cerebrospinal fluid. GEL: gadolinium-enhancing lesion. GFAP: glial fibrillary acidic protein. IgG: immunoglobulin G. IQR: interquartile range. LP: lumbar puncture. MS: multiple sclerosis. OCBs: oligoclonal bands.
* statistically significant with p-value <0.05
a = Kruskal-Wallis, b = Chi-squared test; c = Fisher's exact

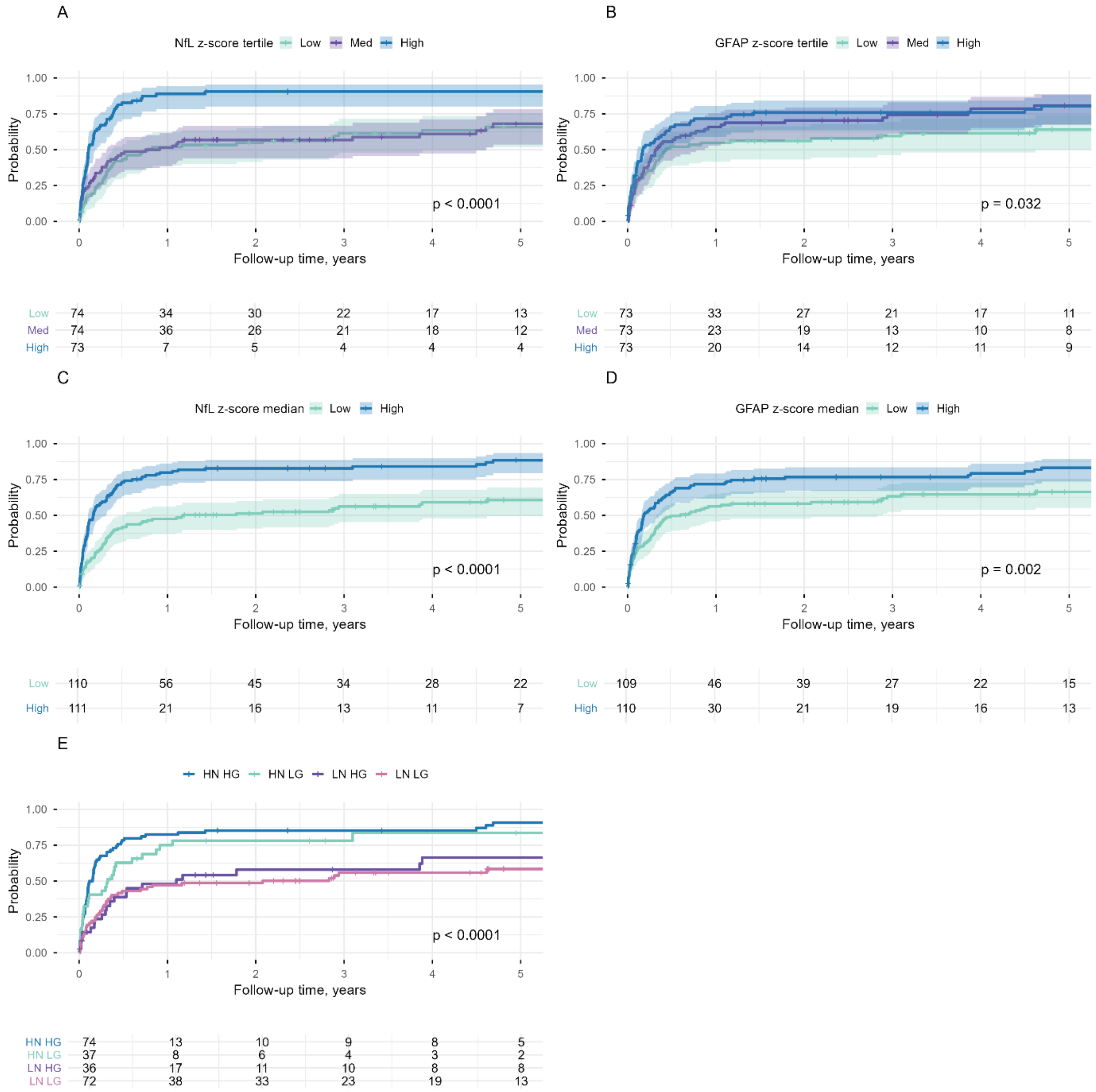

Figure S2 Zoomed-in Kaplan-Meier curves for time to McDonald 2024 MS diagnosis. Zoomed-in view of the Kaplan-Meier curves shown in Figure 2, displaying the identical survival curves with the x-axis restricted to the first 5 years of follow-up to highlight early divergence. Panel A shows the survival analyses of time to McDonald 2024 MS diagnosis by tertiles of NfL, with significant differences between High >2.20 (blue), Medium 1.41-2.20 (purple) and Low ≤1.41 (green). In panel B, time to MS diagnosis is shown for GFAP tertiles, with no significant differences by tertile subgroups (High >0.53 [blue], Medium -0.53-0.53 [purple], Low ≤-0.53 [green]). Panel C and D show the time to MS diagnosis, with NfL (C) divided by median into High ≥1.71 (blue) and Low <1.71 (green), and for GFAP (D) High ≥0.00 (blue) and Low <0.00 (green), with significant differences in both analyses between High and Low. Panel E shows a composite stratification of NfL and GFAP, with faster MS diagnoses in High NfL & High GFAP (blue) and High NfL & Low GFAP (green), compared to Low NfL & High GFAP (purple) and Low NfL & Low GFAP (pink).

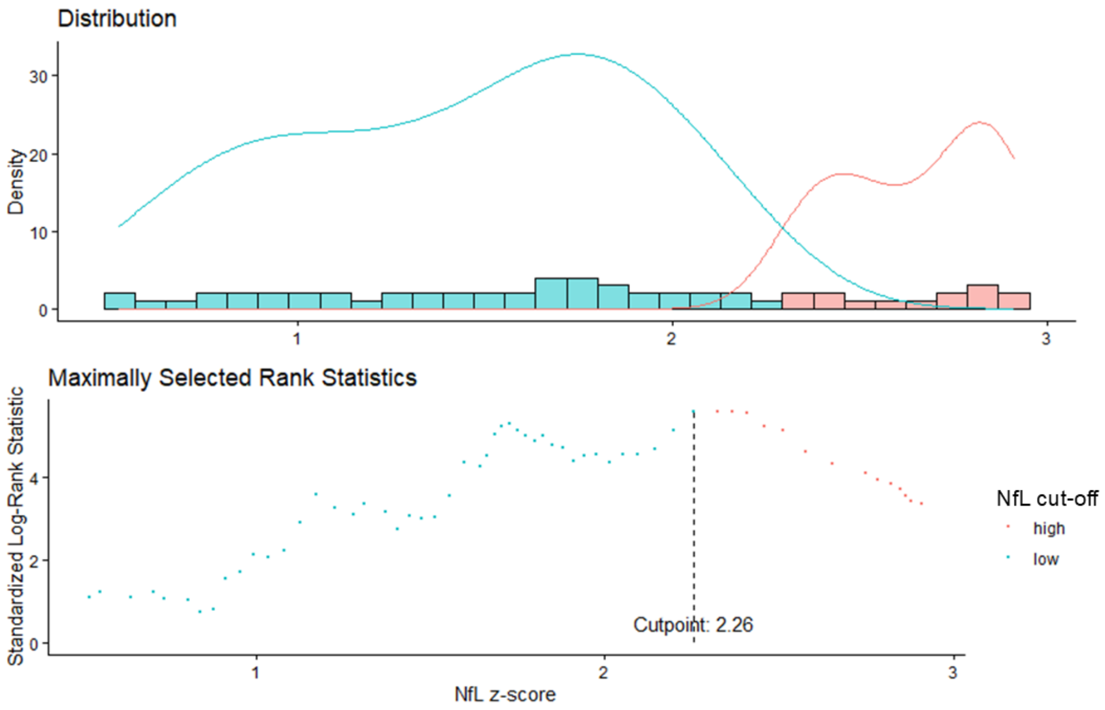

Figure S3 Determination of the optimal cut-off value for NfL z-scores. Using maximally selected rank statistics, the optical NfL z-score cut-off was calculation for our cohort. The vertical dashed line indicates the cut-off (z-score = 2.26) used to classify patients into low (n=159) and high (n=62) NfL groups.

Table S3 | Sensitivity analyses - Cox regression analyses for time to McDonald 2024 MS of MRI and NfL

Model	Variable	HR (95%CI)	p-value	C-index (SE)	LRT χ^2 (p-value)
**MRI-1**	MRI ≥9 T2	3.34 (2.41 – 4.63)	<0.001*	0.672 (0.02)	
**MRI-1+NfL**	MRI ≥9 T2	2.82 (1.99 – 3.98)	<0.001*	0.695 (0.02)	9.64 (0.002*) [a]
	NfL z-score	1.36 (1.12 – 1.66)	0.002*		
**MRI-2**	MRI ≥9 T2	3.15 (2.18 – 4.56)	<0.001*	0.714 (0.02)	
	MRI ≥1 GEL(s)	2.71 (1.88 – 3.91)	<0.001*		
**MRI-2+NfL**	MRI ≥9 T2	2.90 (1.98 – 4.24)	<0.001*	0.720 (0.02)	3.83 (<0.05*) [b]
	MRI ≥1 GEL(s)	2.52 (1.74 – 3.67)	<0.001*		
	NfL z-score	1.25 (1.00 – 1.56)	<0.05*		

Analyses were performed with multivariate Cox proportional hazards models, adjusted for sex and age at CIS. Only complete cases were used, with population in analyses of MRI-1/MRI-1+NfL n=217 and analyses of MRI-2/MRI-2+NfL factors n=195. Additive value of NfL was tested using Likelihood Ratio Test (LTR). Model comparisons: a = MRI-1 vs. MRI-1+NfL. b = MRI-2 vs. MRI-2+NfL.
Abbreviations. CI: confidence interval. CIS: clinically isolated syndrome. GEL: gadolinium-enhancing lesion. HR: hazard ratio. LRT: likelihood ratio test. NfL: neurofilament light chain. SE: standard error.
* statistically significant with p-value <0.05

Table S4 | Additional Cox regression analyses for time to McDonald 2024 MS of NfL, GFAP and risk factors

Model	Variable	HR (95%CI)	p-value	C-index (SE)	LRT χ^2 (p-value)
**NfL, GFAP and risk factors**					
**NfL+wGRS**	NfL z-score	1.34 (1.08 – 1.67)	0.01*	0.691 (0.02)	0.30 (0.58) [a]
	wGRS	1.05 (0.89 – 1.23)	0.58		
**GFAP+wGRS**	GFAP z-score	1.12 (1.02 – 1.21)	0.02*	0.680 (0.02)	0.10 (0.75) [b]
	wGRS	1.03 (0.87 – 1.21)	0.75		
**NfL+GFAP+wGRS**	NfL z-score	1.27 (1.00 – 1.60)	<0.05	0.690 (0.02)	1.77 (0.18) [c]
	GFAP z-score	1.08 (0.97 – 1.20)	0.17		
	wGRS	1.01 (0.87 – 1.20)	0.80		
**NfL+EBV**	NfL z-score	1.35 (1.09 – 1.68)	0.01*	0.689 (0.02)	1.30 (0.25) [d]
	Anti-EBNA1 IgG	1.06 (0.96 – 1.18)	0.26		
**GFAP+EBV**	GFAP z-score	1.13 (1.03 – 1.24)	0.01*	0.679 (0.02)	1.38 (0.24) [e]
	Anti-EBNA1 IgG	1.06 (0.96 – 1.18)	0.25		
**NfL+GFAP+EBV**	NfL z-score	1.27 (1.01 – 1.60)	0.04*	0.686 (0.02)	2.15 (0.14) [f]
	GFAP z-score	1.08 (0.98 – 1.21)	0.13		
	Anti-EBNA1 IgG	1.07 (0.96 – 1.19)	0.24		
**NfL+HLA+EBV**	NfL z-score	1.34 (1.07 – 1.66)	0.01*	0.694 (0.02)	0.58 (0.45) [g]
	HLA-DRB1*15:01	1.41 (0.99 – 2.02)	0.06		
	Anti-EBNA1 IgG	1.04 (0.94 – 1.16)	0.45		
**GFAP+HLA+EBV**	GFAP z-score	1.11 (1.02 – 1.23)	0.03*	0.683 (0.02)	0.70 (0.40) [h]
	HLA-DRB1*15:01	1.28 (0.96 – 1.97)	0.08		
	Anti-EBNA1 IgG	1.05 (0.94 – 1.16)	0.41		

Analyses were performed with multivariate Cox proportional hazards models, adjusted for sex, age and ≥9 T2 lesions on baseline MRI. Anti-EBNA1 IgG titres are log-transformed. Only complete cases were used, with population in analyses of NfL+GFAP n=217 and analyses of NfL+GFAP+risk factors n=189.

Additive value of combined biomarkers was tested using Likelihood Ratio Test (LTR). Model comparisons: a = NfL vs. NfL+wGRS, b = GFAP vs. GFAP+wGRS, c = NfL+wGRS vs. NfL+GFAP+wGRS, d = NfL vs. NfL+EBV, e = GFAP vs. GFAP+EBV, f = NfL+EBV vs. NfL+GFAP+EBV, g = NfL+HLA vs. NfL+HLA+EBV and h = GFAP+HLA vs. GFAP+HLA+EBV.

Abbreviations. CI: confidence interval. CIS: clinically isolated syndrome. EBNA1: Epstein Barr virus nuclear antigen-1. EBV: Epstein Barr virus. GFAP: glial fibrillary acidic protein. HLA: human leukocyte antigen. HR: hazard ratio. IgG: immunoglobulin G. LRT: likelihood ratio test. NfL: neurofilament light chain. Ref: reference. SE: standard error. wGRS: weighted genetic risk score.

* statistically significant with p-value <0.05
